# The Aetiopathogenesis of Late Inflammatory Reactions (LIRs) After Soft Tissue Filler Use: A Systematic Review of the Literature

**DOI:** 10.1007/s00266-021-02306-3

**Published:** 2021-04-28

**Authors:** Y. Bachour, J. A. Kadouch, F. B. Niessen

**Affiliations:** 1 Department of Plastic, Reconstructive and Hand Surgery, Amsterdam UMC-location VUmc, De Boelelaan 1117, PO Box 7057, 1007 MB Amsterdam, The Netherlands; 2 Department of Dermatology, ReSculpt Clinic, Amsterdam, The Netherlands

**Keywords:** Filler, Soft tissue filler, Dermal filler, Complications, Adverse events, Late inflammatory reactions, Aetiopathogenesis

## Abstract

**Background:**

Late inflammatory reactions (LIRs) are the most challenging complications after filler use. The immune system plays a prominent role in its etiology, albeit to an unknown extent. Bacterial contamination in situ has been hypothesized to be causative for LIRs. How this relates to the immunological processes involved is unknown. This article aims to provide an overview of immunological and bacterial factors involved in development of LIRs.

**Methods:**

We undertook a systematic literature review focused on immunological factors and microbiota in relation to LIRs after filler use. This systematic review was performed in accordance with the PRISMA guidelines. PubMed, EMBASE and the Cochrane databases were searched from inception up to August 2019. Included studies were assessed for the following variables: subject characteristics, number of patients, primary indication for filler injection, implant type/amount and injection site, type of complication, follow-up or injection duration, study methods, type of antibiotics or medical therapies and outcomes related to microbiota and immunological factors.

**Results:**

Data on immunological factors and bacterial contamination were retrieved from 21 included studies. Notably, the presence of histocytes, giant cells and *Staphylococcus epidermidis* within biopsies were often associated with LIRs.

**Conclusion:**

This review provides a clear overview of the immunological factors associated with LIRs and provides a hypothetical immunological model for development of the disease. Furthermore, an overview of bacterial contamination and associations with LIRs has been provided. Follow-up research may result in clinical recommendations to prevent LIRs.

**Level of Evidence III:**

This journal requires that authors assign a level of evidence to each article. For a full description of these Evidence-Based Medicine ratings, please refer to the Table of Contents or the online Instructions to Authors-www.springer.com/00266..

**Supplementary Information:**

The online version contains supplementary material available at 10.1007/s00266-021-02306-3.

## Introduction

Fillers, also referred to as dermal fillers, soft tissue fillers or dermal implants have been used for decades, mainly for cosmetic reasons, but also to improve aesthetic outcome after trauma, cancer or malformations [1]. They are bioinjectable materials that are approved by the FDA as medical devices or implants. Soft tissue fillers are injected transcutaneously through a needle or cannula into/or between the dermis and subcutaneous fat. It is a noninvasive surgical procedure that must be performed in a setting where preoperative antiseptic requirements are taken into account [2]. Over the past decade, their use for aesthetic purposes has increased exponentially. With more than 2.5 million procedures in 2018, it has been the most frequent non-surgical procedure in plastic surgery in the USA [3]. The increase of their use has subsequently resulted in an increase of soft tissue filler products. Although there is no universally accepted classification for these products, they are mostly classified by their biodegradability into temporary-, biostimulatory- or permanent fillers [4, 5]. Biostimulatory fillers (formerly referred to as semi-permanent) exert their definitive filling effect indirectly by inducing volumization through neocollagenesis stimulation at the site of injection [4]. An overview of the most used (in present and past) soft tissue fillers according to this classification can be found in Table 1.

**Table 1:** Overview of dermal implants

Biodegradability/longevity	Substances	Brand names	Duration of effect
Temporary	Collagen (not used anymore), hyaluronic acid	Restylane, Juvéderm, Belotero	6–24 months
Biostimulatory	Polylactic-L-Acid (PLA), calcium hydroxylapatite (CHA), polycaprolactone	Radiesse, Sculptra Ellansé	12–36 months
Permanent	Silicone, polyalkylimide gel (PAIG, Bio-Alcamid), polyacrylamide gel (PAAG, Aquamid), polymethyl-methacrylate (PMMA, Artocoll/ArteFill), HEMA/EMA (DermaLive)	Artefill, Dermalive, Aquamid, Bio-Alcamid	–

An overview of the different dermal implants according their biodegradability. This concerns the longevity of the dermal implant after injection and varies from temporary, to biostimulatory and permanent implant

**Fig. 1:**
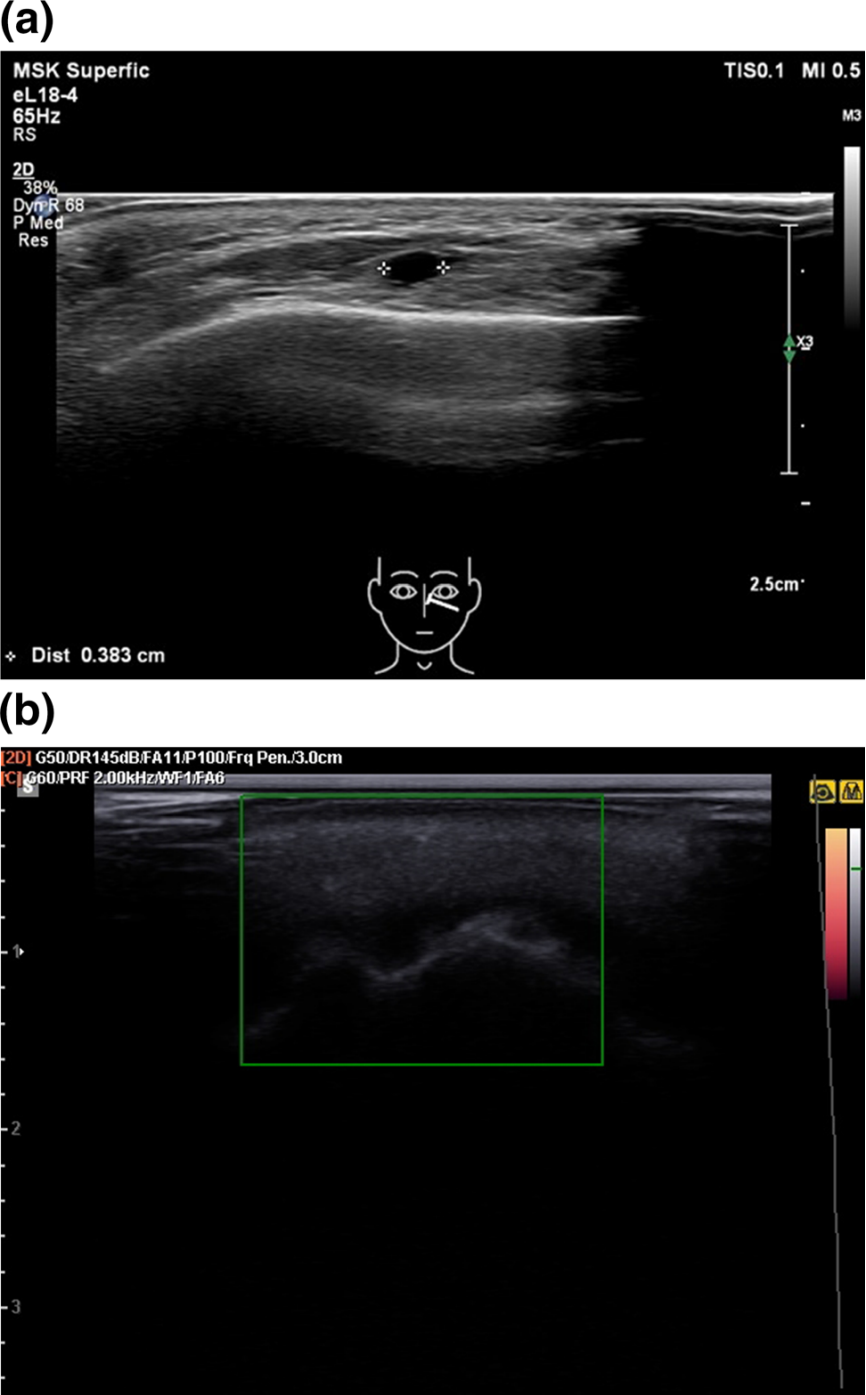
Ultrasound images of a filler depots. **a** On this ultrasound image of the tear trough, a 40mm sized anechoic (black) process is seen, which matches the image of hyaluronic acid filler. **b** On this ultrasound image of the cheek area of a patient injected with a calcium hydroxylapatite filler. We see a cloudy isoechoic cloudy depot with multiple hyperechoic dots (=calcium hydroxylapatite particles), preventing ultrasound penetration good definition of underlying anatomical structures

Although manufacturers and several studies claim that adverse events after dermal fillers are very uncommon, unwanted adverse events do occur with all fillers products (Fig. 1a, b) [6–12]. A review by de Vries et al. shows more specifically that adverse events have been reported for almost every fillers product [13]. Of these, late filler reactions such as lumps, nodules, swellings or granulomas are the most challenging to treat since their exact aetiopathology is unknown [7]. Inflammatory nodules usually emerge between several weeks up to several years after the procedure and are therefore also called late inflammatory reactions (LIRs) [14]. It is estimated that these complications occur in 0.01%–0.1% of the procedures that have been carried out [15]. Moreover, especially the permanent filler-induced LIRs, often cause a permanent disfigurement of the faces of treated patients [4, 9, 16, 17].

A foreign body reaction is a natural tissue response after soft tissue filler injection [18]. The immune response around the filler material involves macrophages, histiocytes, lymphocytes and giant cells [10, 12, 19]. In time, these cells reduce in numbers and chronic inflammation cells accumulate around and within the filler material [5]. An inflammatory nodule may then be formed [20]. To date, the aetiopathology of LIRs is unknown and there is much debate about the pathogenesis of this disorder. Many authors have focused on injection techniques and types of filler products in relation to the development of LIRs [21, 22]. However, adaptation of filler characteristics has not significantly diminished the incidence of LIRs. Several risk factors have been identified, and additional causative factors have been proposed, such as occurrence of a subclinical infection, an excessive foreign body reaction and biodegradability of the filler material [15, 18, 23, 24]. Although none of these theories are conclusive so far, there is consensus that the immune system plays a prominent role in the development of LIRs, albeit to an unknown extent [25–27].

Bacterial contamination of the filler material has frequently been proposed as a likely cause of LIRs and some indications for this theory exist [7, 8, 28]. To understand the possible role of contamination, a clear picture of the immunopathogenesis of LIRs is necessary. Therefore, the aim of this systematic review is twofold: (1) to give an overview of immunological factors involved in LIRs, and (2) to determine the role of bacterial contamination in LIRs. Although nowadays most permanent fillers are banned for cosmetic use, complications following their injection in the past are still seen in current clinical practice. Therefore, we have chosen to include them the literature search and review for this article.

## Materials and Methods

### Literature Search

A systematic review was performed of the literature pertaining to immunological factors and bacterial contamination/infection of LIRs after soft tissue filler use. This systematic review was carried out in accordance with the Preferred Reporting Items for Systematic Reviews and Meta-Analyses Statement (PRISMA) guidelines [29]. An extensive search was conducted using the electronic databases PubMed, Embase.com and Wiley/Cochrane Library. Appropriate keywords in the English language were combined by Boolean logical operators and adapted to the appropriate syntax of each database. The following terms were used (including synonyms and closely related words) as index terms or free-text words: ‘adverse events’ and ‘filler.’ The full search strategies for all the databases can be found in the Supplementary Information. Studies written in English and Dutch from September 1975 until August 2019 were reviewed.

### Selection Criteria

We searched for original studies on LIRs after soft tissue filler use which reported on microbiota and/or immunological factors. All patient studies as well as relevant animal studies were included. Of these studies, all types of filler products were included, as well as all sites of injection. In vitro studies and studies which investigated the normal immune response of filler products were excluded. Case reports less than 5 patients and isolated abstracts were excluded as well as non-original studies such as reviews, editorials, communications, correspondence, discussions and letters.

The search was executed in cooperation with a medical information specialist. The article selection was performed by two reviewers in two steps: in the first step eligibility of the articles was screened based on the title. Subsequently, the abstracts of the selected articles were evaluated, and, in case of doubt, the full article was reviewed. Articles which met the inclusion criteria were included in the systematic review.

### Data Collection and Analyses

Data were extracted using standardized tables developed for this purpose. Data included the following variables: authors, year of publication, study design, number of patients or samples, type/amount and location sites of injected filler, included group of subjects, primary indication for injection, type of complication, follow-up or injection duration, microbial/immunological methodologies, type of antibiotics/medical therapies, main outcomes and the conclusion. Included studies were assigned a level of evidence according to the Oxford Centre for Evidence-Based Medicine [30].

Due to heterogeneity of the studies, statistical meta-analysis of the data is impossible. Instead, we performed qualitative and descriptive analyses of the outcomes.

## Results

### Article Demographics

**Fig. 2:**
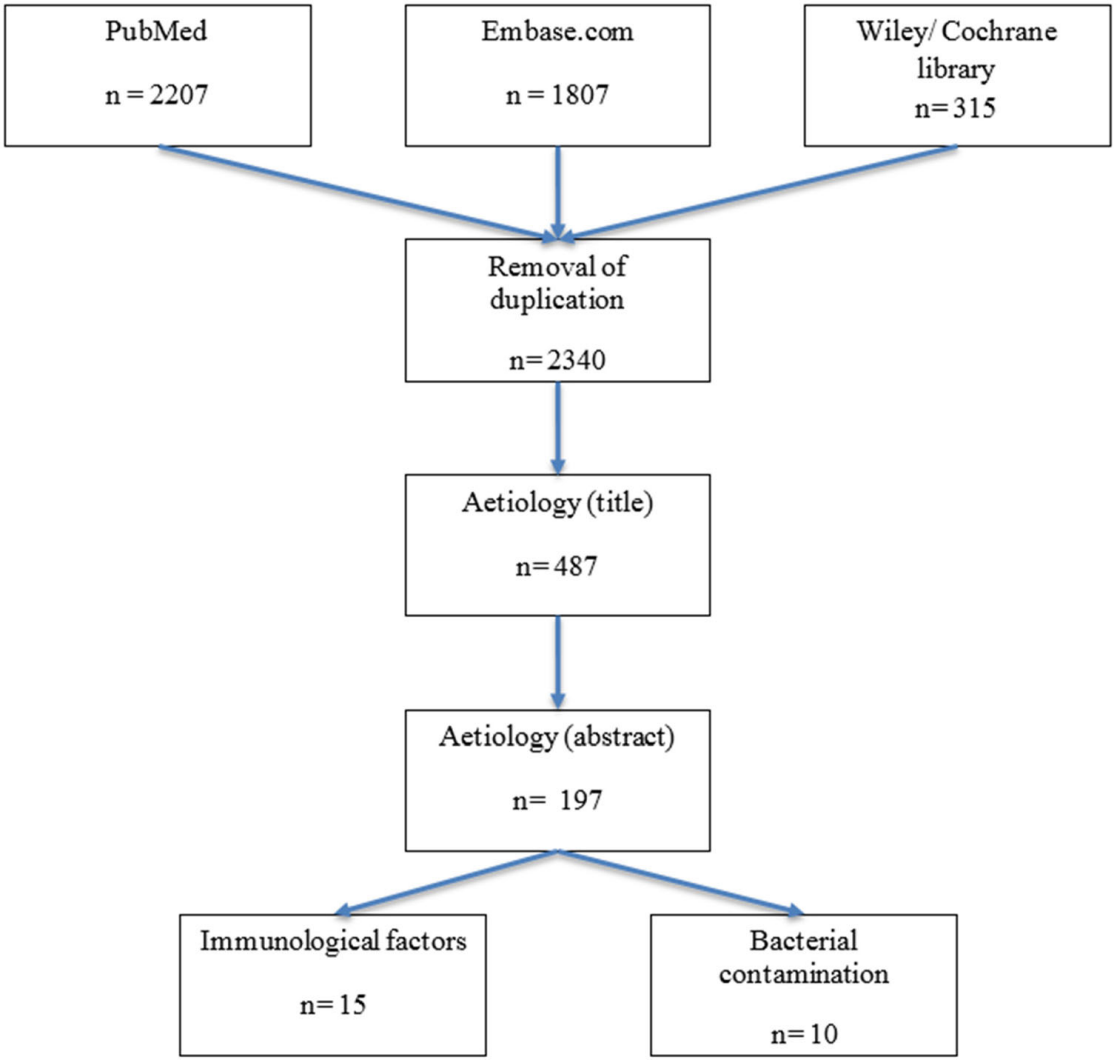
Study selection flowchart

The literature search produced 2340 articles after removal of duplicates. Of these, 487 remained after title screening, and 197 after evaluation of the abstracts. Full text review yielded 21 studies which met the inclusion criteria (Fig. 2). Studies were classified into two major subject groups: (1) immunological factors and (2) bacterial contamination. The baseline characteristics of included studies are given in Table 2.

**Table 2:** Characteristics of included studies

Author	Publication year	Study design	N Patients (dermal implant/tissue/biopsies)	Type of implant	Amount of injected implant	Injection site	Main focus	Level of evidence
Alijotas-Reig et al. [32]	2012	Case series	7 (Unknown)	Permanent	Unknown	Various	Immunology (local and systemic)	3b
Christensen et al. [31]	2005	Cross-sectional study	Unknown (33)	Permanent	Unknown	Various	Immunology (local)	3b
Daley et al. [26]	2012	Case series	8 (Unknown)	Biostimulatory	Unknown	Mandibular labial vestibule, lips	Immunology (local)	4
de Melo Carpaneda et al. [11]	2012	Case- control study	96 (Unknown)	Permanent	Unknown	Various	Immunology (local)	3b
El-Khalawany et al. [33]	2015	Case series	38 (Unknown)	Temporary, biostimulatory and permanent	Unknown	Various	Immunology (local)	4
Eversole et al. [24]	2013	Case series	12 (Unknown)	Temporary, biostimulatory and permanent	Unknown	Lips	Immunology (local)	3b
Ficarra et al. [25]	2012	Case series	7 (Unknown)	Permanent	Unknown	Lips	Immunology (local)	4
Kadouch et al. [9]	2015	Case series	19 (21 biopsies)	Biostimulatory and permanent	Unknown	Various	Immunology (local)	4
Lombardi et al. [34]	2004	Case series	11 (Unknown)	Biostimulatory and permanent	Unknown	Various	Immunology (local)	4
Micheels et al. [10]	2001	Cross-sectional study	8 (Unknown)	Short-acting and biostimulatory	Unknown	Unknown	Immunology (local and systemic)	3b
Owosho et al. [4]	2014	Case series	16 (Unknown)	Biostimulatory and permanent	Unknown	Various	Immunology (local)	4
Rudolph et al. [17]	1999	Case series	5 (Unknown)	Short-acting and biostimulatory	Unknown	Face and nipple	Immunology (local)	4
Shahrabi-Farahani et al. [1]	2014	Case series	25 (Unknown)	Biostimulatory	Unknown	Various	Immunology (local)	4
Wiest et al. [18]	2009	Case series	10 (Unknown)	Permanent	Unknown	Various	Immunology (local)	4
Alijotas-Reig et al. [32]	2012	Cross-sectional study	7 (Unknown)	Permanent	Unknown	Various	Bacterial contamination	3b
Bjarnsholt et al. [27]	2009	Case series	8 (Unknown)	Permanent	Unknown	Various	Bacterial contamination	4
Christensen et al. [6]	2013	Cross-sectional study	78 (Unknown)	Permanent	Unknown	Face and lips	Bacterial contamination	3b
Christensen et al. [31]	2005	Cross-sectional study	Unknown (33 biopsies)	Permanent	Unknown	Various	Bacterial contamination	3b
Kadouch et al. [7]	2013	Cross-sectional study	Unknown (18 biopsies)	Permanent	Unknown	Various	Bacterial contamination	3b
Netsvyetayeva et al. [23]	2018	Cross-sectional study	27 (Unknown)	Biostimulatory	1-3 mL	Various	Bacterial contamination	3b
Saththianathan et al. [30]	2017	Cross-sectional study	5 (6 biopsies)	Biostimulatory and permanent	Unknown	Unknown	Bacterial contamination	3b
Wiest et al. [18]	2009	Case series	10 (Unknown)	Permanent	Unknown	Various	Bacterial contamination	4
Alijotas-Reig et al. [32]	2012	Cross-sectional study	7 (Unknown)	Permanent	Unknown	Various	Anti-inflammatory	3b
de Melo Carpaneda et al. [11]	2012	Case- control study	96 (Unknown)	Permanent	Unknown	Various	Anti-inflammatory	3b
Alhede et al. [49]	2014	Animal study	48 (Unknown)	Biostimulatory and permanent	100 mcg	Unknown	Anti-inflammatory	n/a
Bjarnsholt et al. [27]	2009	Case series	8 (Unknown)	Permanent	Unknown	Various	Antibiotics (systemic)	4
Marusza et al. [13]	2019	Cross-sectional study	22 (Unknown)	Biostimulatory	2.09 ml (range 1-6 ml)	Various	Antibiotics (systemic)	3b
Alhede et al. [49]	2014	Animal study	48 (48 Unknown)	Biostimulatory and permanent	100 mcg	Unknown	Antibiotics (systemic)	n/a

### Bacterial Contamination

Eight human studies (including 168 patients) have investigated the presence of bacteria on samples from LIRs after filler use [7, 8, 19, 23, 28, 31–33]. Six studies were cross-sectional studies, while two studies were case series. Furthermore, two human study (including 30 patients) and one animal study investigated the effect of systemic antibiotics for the treatment of LIRs to prove the role of bacterial contamination in the aetiopathogenesis of filler-related LIRs. These were a cross-sectional study and a case series.

### Immunological Factors

We found 14 studies with regard to a variety of immunological factors in relation to LIRs [1, 5, 10–12, 18, 19, 25–27, 32–35]. These studies with a total of 295 patients described local presence of immune cells or immunological factors using histological or histochemical techniques. Eleven studies were case series, two were cross-sectional studies, while one study was a case-control study. Systemic immunological factors were reported in 2 studies including 15 patients in total. Two studies reported on the systemic as well as on the local presence of immune cells. Several groups investigated whether local and/or systemic immunological factors were associated with adverse events, by investigating the role of medical therapies. Two human studies (one cross-sectional study and one case-control study) and one animal study used medical therapies for preventing or diminishing LIRs.

## Discussion

### Bacterial Contamination and LIRs

The exact aetiopathology of LIRs caused by filler is still poorly understood. Several authors have suggested that delayed-onset complications can occur in response to local or systemic infections, (facial) injuries, systemic medication or vaccinations, or invasive treatments (such as dental surgery) in the vicinity of the filler deposits [8, 36–44].

Numerous human studies have shown the presence of bacteria in material derived from patients with LIRs (Table 7 in ESM) [7, 8, 23, 28, 31, 32]. Two studies did not detect any bacteria in the collected samples [19, 33]. The most common detected bacteria were *Staphylococcus spp.* (mainly *Staphylococcus epidermidis*) and *Propionibacterium acnes*, which were detected in up to 98% of positive cultures, and which lead to the hypothesis that the presence of these bacteria is involved in development of LIRs [7, 8, 24, 28, 31, 32]. Two studies compared bacterial presence in samples from patients with adverse events and patient with no adverse events, both finding a much higher rate of bacteria in the adverse events’ group [7, 24].

In a human study by Christensen et al., they showed that bacterial presence varies among different filler compounds, with an increased contamination within the permanent filler material [32]. This finding has been supported by one animal study by Alhede et al. This experiment left contaminated gels in a mouse model finding a sustainment of bacterial growth within the permanent gel, less in the biostimulatory gel and no growth within the temporary gel [45]. The latter might explain the difference in complication rate between permanent, biostimulatory and temporary filler material.

Despite this evidence for the presence of bacteria around filler material and support for a primary role in the development of LIRs, some authors postulate that these findings are not enough to conclude LIRs have an infectious aetiopathology [46]. They state that the proof of such a conclusion cannot be based on the visualization of a few bacteria on histologic slides or by genetically identifying them, but to demonstrate the presence of a biofilm formation with hundreds of bacteria.

### Immunological Factors Associated with LIRs

**Fig. 3:**
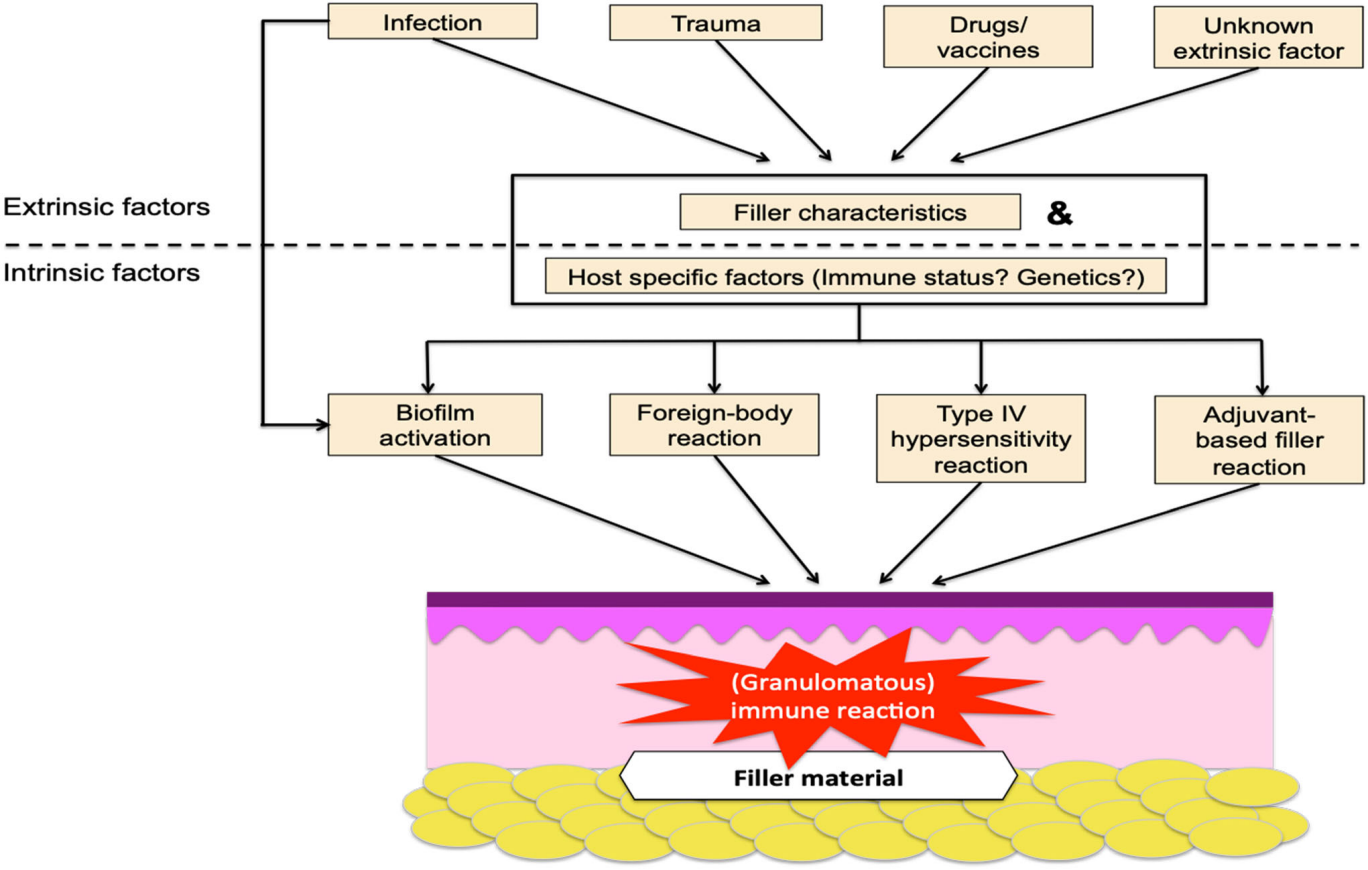
Extrinsic and intrinsic factors in the development of delayed-onset complications of fillers Late inflammatory reactions of injected filler usually considered to be a result of foreign body reactions, microbial contamination (in biofilms or otherwise) of filler material, type IV hypersensitivity reactions or adjuvant-based filler reactions. Although the underlying pathomechanisms are largely unresolved, they are probably influenced by host specific factors, such as immune status and genetic background, as well as by characteristic of the filler material itself

Multiple studies indicate that extrinsic factors such as (micro-) trauma, infections and drugs or vaccinations seem to be capable of activating certain mechanisms leading to delayed-onset complications such as (low-grade) inflammatory nodules and abscess formation (Fig. 3). Although the exact underlying mechanisms responsible for eliciting such complications remain unknown, filler characteristics and host immune status both appear to play a role [8, 37, 47–50]. In our literature search, we found that several studies have evaluated the role of immunological factors in the development of LIRs and show involvement of several cell types inducing inflammatory processes (Figure 3, Table 6 in ESM) [1, 5, 10–12, 18, 19, 25–27, 32–35]. Namely (epithelioid) macrophages, histiocytes, lymphocytes, giant cells were demonstrated HE stained sections of these filler nodules. In some cases also, neutrophils, eosinophils or (plasmacytoid) dendritic cells (DCs) were found.

#### Foreign Body Reactions

The generation of a granulomatous foreign body reaction (GFBR) is a ‘normal,’ physiological response from the host to any foreign body. All filler agents used for soft tissue augmentation are thought to elicit some degree of granulomatous inflammatory reaction following injection [49, 51]. To a certain point, this is considered to be part of a normal physiological response [49, 52]. GFBR can be classified according to a severity grading system and/or into different clinicohistological phenotypes. Duranti proposed a 4-point grading system for the severity of GFBR [20]. Lemperle and Lombardi proposed a classification of GFBR into different clinical and/or histological subtypes [35, 49]. A genuine GFBR is predominantly composed of histiocytes/macrophages and giant cells encapsulating filler particles [20, 49]. The exact pathophysiology of filler-induced GFBR, or ‘filler granulomas,’ has yet to be elucidated.

Current insights differentiate several steps in host reaction on biomaterial implantation. Within minutes after implantation, host plasma components such as albumin, fibrinogen, fibronectin, lipids, sugars, ions and platelets are absorbed on the surface of the implant [7, 22, 53]. Platelets and other components of the coagulation cascade induce clot formation, which also functions as the provisional matrix around the biomaterial. Platelet adhesion and activation, and the release of pro-inflammatory cytokines, chemokines and growth factors induce sequentially the acute and chronic inflammatory responses on the implanted material. Damage-associated molecular patterns (DAMPs), pathogen-associated molecular patterns (PAMPs) and Alarmins present at the implantation site activate macrophages, leukocytes and dendritic cells through pattern recognition receptors (PRRs), toll-like receptors (TLRs) and C-type lectin [45–47, 53–55]. In the acute phase, the recruited neutrophils attempt to degrade the biomaterial through phagocytosis and the release of proteolytic enzymes, reactive oxygen species (ROS) and neutrophil extracellular traps (NETs, consisting of granular proteins, elastase, histones and chromatin DNA) [50, 51, 53]. NETs are usually involved in trapping pathogens and prevention of infection spread.

The release of immune-regulatory signals by neutrophils attracts monocytes, macrophages, immature DCs and lymphocytes, promotes monocytes differentiation into ‘M1’-type macrophages secreting pro-inflammatory cytokines (Interleukin (IL-) 1β, IL-6, necrosis factor alpha (TNF-α)) and downregulates the presence and activity of neutrophils themselves. The next step in the healing process is a shift toward ‘M2’-type macrophages secreting anti-inflammatory cytokines (i.e., IL-10) and recruiting fibroblasts for effective tissue regeneration. The shift toward an anti-inflammatory ‘M2’-type healing response can be altered by specific characteristics of the implanted material. For example, particles larger than 5 μm require the presence of aggregated macrophages, or giant cells, to be phagocytosed [56–58]. Particles larger than 15 to 20 μm are generally not subject to ingestion by macrophages by true phagocytosis, but may be enclosed by giant cells. Failure of effective phagocytosis leads to a chronic inflammation pathway and granuloma formation, consisting of macrophages and giant cells, as well as a contiguous infiltrate of lymphocytes secreting pro-inflammatory cytokines (i.e., tumor necrosis factor alpha (TNF-α), interferon gamma (IFN-γ) and interleukin 12 (IL-12)) [43, 53]. Similarly, excessive production of NETs by neutrophils can impair healing and lead to a chronic inflammation and encapsulation [53, 57, 58].

Numerous studies have been performed on the different structural properties of filler, such as chemical composition, electrical charge, surface irregularities or particle size and the presence of contaminants, which are of known influence on host immune responses [49, 50] (Fig. 3, Table 3).

**Table 3:** Chemical properties of dermal implants and their immunological effect [56]

Structural properties	Immunological effect
Electrical charge	
Positively charged particles	Attract and/or activate macrophages
Negatively charged particles	Repel some negative charged bacteria
Surface irregularities	
Irregular surfaces	Elicit a longer-lasting inflammatory reaction
Smooth surfaces	Formation of a thicker fibrous capsule around the material
Particle size	
Large particles (>20 μm)	No phagocytosis
Small particles (<20 μm)	Fast phagocytosis, resulting in a greater local inflammatory reaction
Hydrophilic/hydrophobic	
Hydrophilic polymer gels	Highly biocompatible and easily penetrable by nutrients and waste products
Hydrophobic polymer gels	Favor fibronectin absorption and therefore cellular adhesion, thus promoting a pro-inflammatory response
HA implants (molecular size and the amount of chemical cross-linking)	
Low molecular weight HA	Acts pro-inflammatory and triggers the immune system
High molecular weight HA	Acts anti-inflammatory properties

The relation with time can also be of importance. Most studies show a decreased inflammatory reaction after several months; however, this also depends on the type of filler used [32]. Whereas material from acute lesions (up to 30 days) shows a cellular infiltrate composed mainly of neutrophils, lymphocytes, macrophages and cells similar to fibroblasts, tissues from 6 months on show predominantly the presence of small and round empty spaces surrounded by dense and organized inflammatory and fibrous tissue. In some cases, capsule formation was reported.

#### Delayed-Type Hypersensitivity Reactions

Immune-mediated hypersensitivity to fillers in patients with LIRs has been investigated by one study. Micheels et al. showed that skin tests in patients with adverse events were positive for one or the other or both of the injectable hyaluronic acid preparations [11]. Also, serum analysis revealed positive antibodies against Restylane and/or Hylaform and even IgG and E anti-hyaluronic acid. Delayed hypersensitivity reactions are often proposed in the literature as cause for LIRs. Some authors have in fact postulated that all granulomatous reactions to fillers are in fact type IV (delayed-type) hypersensitivity reactions (Fig. 3) [59]. However, it must be noted that a true type IV hypersensitivity reaction is a systemic immune response that should affect all injected sites at the same time [23, 60]. In addition, to our knowledge non-autologous (bovine) collagen and hyaluronic acid are the only two main filler constituents reported to be capable of eliciting type IV hypersensitivity reactions, substantiated by positive skin-testing [11, 17, 61, 62]. Bovine collagen is the carrier for PMMA fillers, whereas hyaluronic acid has by far been the most widely used temporary filler this past decade. Although, T cell-mediated delayed-type hypersensitivity reactions to these constituents could play a role in LIRs to these two filler types, have not found any literature, other than the paper by Micheels et al., supporting this hypothesis with golden standard allergic testing.

#### Adjuvant-Based Filler Reactions

Alijotas-Reig recently postulated that fillers may act as adjuvants, rather than as antigens [37]. Adjuvants are defined as substances that may stimulate immune responses without having specific antigenic properties themselves [63]. Both the innate and adaptive immune systems are influenced by the effects of adjuvants. Adjuvants enhance innate immune responses by mimicking evolutionary conserved molecules (e.g., PAMPs) capable of binding toll-like receptors (TLRs, mainly activating TLRs 1, 4, 5, 7 and 9), resulting in the release of T_h_1 inflammatory cytokines [63]. They also increase the activity of dendritic cells (DCs), lymphocytes and tissue macrophages. The immune-enhancing effects of adjuvants are supposedly mediated by five different activities, as depicted in Table 4 [63, 64]. Certain triggers such as infection, trauma and vaccination may induce adjuvant activity or act as adjuvants themselves [37, 63–66]. In addition, sequential exposure to different adjuvant stimuli is believed to increase the risk of abnormal immune responses [37, 66]. Several studies on animals and humans have demonstrated that adjuvants are able to induce autoimmunity and autoimmune diseases [63]. Recently, the name ‘autoimmune/inflammatory syndrome induced by adjuvants,’ in short ‘ASIA’ or Schoenfeld’s syndrome, was introduced to describe the spectrum of immune-mediated systemic diseases that may be triggered by previous exposure to an adjuvant stimulus [67, 68] (Table 5). Several studies have demonstrated similar systemic inflammatory responses after the use of filler [37, 47, 48, 59, 65, 69–71]. Yet unknown factors, related to the specific adjuvant(s) involved and to the extent in which innate, adaptive and regulatory immune responses are activated, are believed to determine whether an autoimmune response remains limited or will evolve into full-blown systemic disease [37, 47, 48]. Genetic predisposition for the development of ASIA is also suspected [37, 47, 66]. Known adjuvants that may cause ASIA are silicone, aluminum salts, pristane and infectious components [63, 65, 67]. In addition, hyaluronic acid compounds and acrylamides have also been identified as adjuvants [16, 37, 47, 48, 71, 72]. More researched is needed to support and further establish this hypothesis.

**Table 4:** Adjuvant immunological effect exerted by different modes of action [63]

No.	Mode of action	Immunological effect
1.	Translocation of antigens to the lymph nodes where they can be recognized by T cells	Enhancing T cell activity, increased clearance of pathogen throughout the organism
2.	Protection of antigens, which grants the antigen a prolonged delivery and exposure	Upregulating the production of B and T cells needed for enhanced immunological memory as part of the adaptive immune response
3.	Increasing the capacity to cause local reactions at the injection site	Greater release of danger signals by chemokine releasing cells such as T cells and mast cells
4.	Inducing the release of inflammatory cytokines	Recruitment of B and T cells at sites of infection and increased transcriptional events leading to a net increase of immune cells as a whole
5.	Interacting with pattern recognition receptors (PRRs), specifically toll-like receptors (TLRs), on accessory cells	Increase of the innate immune response to antigen

**Table 5:** Criteria for the diagnosis of ASIA* as suggested by the group of Dr. Shoenfeld [63]

*Major criteria*
Exposure to an external stimulus (infection, vaccine, silicone, adjuvant) prior to clinical manifestation of symptoms
The appearance of ‘typical’ clinical manifestations:
Myalgia, myositis or muscle weakness
Arthralgia and/or arthritis
Chronic fatigue, unrefreshing sleep or sleep disturbances
Neurological manifestations
Cognitive impairment, memory loss
Pyrexia, dry mouth
Removal of an inciting agent induces improvement
Typical biopsy of involved organs
*Minor criteria*
The appearance of autoantibodies or antibodies directed at the suspected adjuvant
Other clinical manifestations (i.e., irritable bowel syndrome)
Specific HLA haplotypes (i.e., HLA DRB1, HLA DBQ1)
Evolvement of an autoimmune disease (i.e., MS, SSc)

### Medical Therapies

Anti-inflammatory medications have been investigated in several studies as possible therapy for LIRs (Table 8a in ESM) [12, 14, 28, 33, 45]. Although some studies are promising, contradictive results exist [12, 14, 28, 33, 45]. Tacrolimus has been used as an immunosuppressant drug in transplantations and granulomatous disease [73]. Alijotas-Reig et al. investigated its use in series of seven patients with late-onset adverse events after silicone injection, finding good clinical response [33]. De Melo Carpaneda et al. investigated in a series of seven patients the effect of intralesional corticosteroids [12]. These patients reported an initial recovery with softening of the compromised region, but after a few months, the nodule became harder and the skin of the region where the product had been injected turned whitish and depressed indicating corticosteroid induced atrophy. Alhede et al. investigated in their animal model the effect of triamcinolone acetonide in combination with antibiotics [45]. They contaminated gels and left them for seven days in a mouse model. This study showed that once the bacteria had settled (into biofilms) within the gels, even successive treatments with high concentrations of relevant antibiotics/corticosteroids were not effective.

Also an antimicrobial approach for the treatment of LIRs has been widely addressed (Table 8b in ESM). The hypothesis stating bacteria in situ are involved in the development of adverse events is further investigated by Marusza et al. who treated patients with an antibiotic scheme of whom all experienced resolution of symptoms and with no recurrence of their complication [14]. However, these results must be interpreted with care, as the used antibiotic (clarithromycin) is also known for its immune modulating properties and the used treatment scheme also included intralesional hyaluronidase injections which dissolves the filler. Therefore, it is not possible to draw conclusions based on this study.

Although a lot has been written on the best treatment approach for LIRs, to date no evidence-based effective therapy for LIRs exists. To achieve this, first the aetiopathological uncertainties most be solved, keeping in mind that maybe not all LIRs have the same aetiopathology or cause. Also, although bacterial presence might not necessarily prove an infectious process, bacterial contamination of the filler material might act as stimulus for other (innate) immune response. This is why many practitioners use and will keep using immune modulating antibiotics as first line therapy in case of HA-induced LIRs. This way treats both an infectious and an inflammatory reaction.

The best approach, however, remains prevention. For this reason, possible causes or triggers for an infectious or inflammatory process should be avoided. Filler treatments should therefore be performed in under aseptic conditions by well-trained physicians with knowledge of skin preparation, facial anatomy and potential complications. Like described by De Boulle and Heydenrych, special considerations are necessary for patients with preexisting autoimmune or -inflammatory diseases [17].

It must also be empathized that (chemical properties of) permanent fillers have been proven to have a significantly higher risk for adverse events such as bacterial contamination, inflammatory reactions, abscesses and migration up to decades after injection. Their use should therefore be discouraged, especially for aesthetic indications.

#### Recommendations for Injection Techniques and Treatment Options

As previously mentioned, dermal fillers can be used for cosmetic but also for medical reasons. For both indications, there might be a loss of elasticity and collagen in the dermis, volume loss of the subcutaneous fat and bone structures. As a result of this, injection of fillers needs a three-dimensional approach. The injection of fillers is therefore divided into three injection levels: deep dermal, subdermal and supraperiosteal [74]. Although there is not a clear algorithm for the injection of fillers in the tissue, there is a consensus for the following injection techniques for different types of fillers; HA skinboosters should be injected into the dermis using a thin needle. Biostimulatory and HA fillers could be injected subdermal with the use of a cannula using the fanning technique. However, biostimulatory fillers should not be used in the periorbital and perioral area as there are muscular dynamics that cause a movement of the injected filler. Biostimulatory and HA fillers can be injected on the periost using a needle. Here, it is advised to inject low volume of the compound (around 0,025 mL, the so called ‘JVL bolus’) in order to prevent any vascular adverse events.

**Fig. 4:**
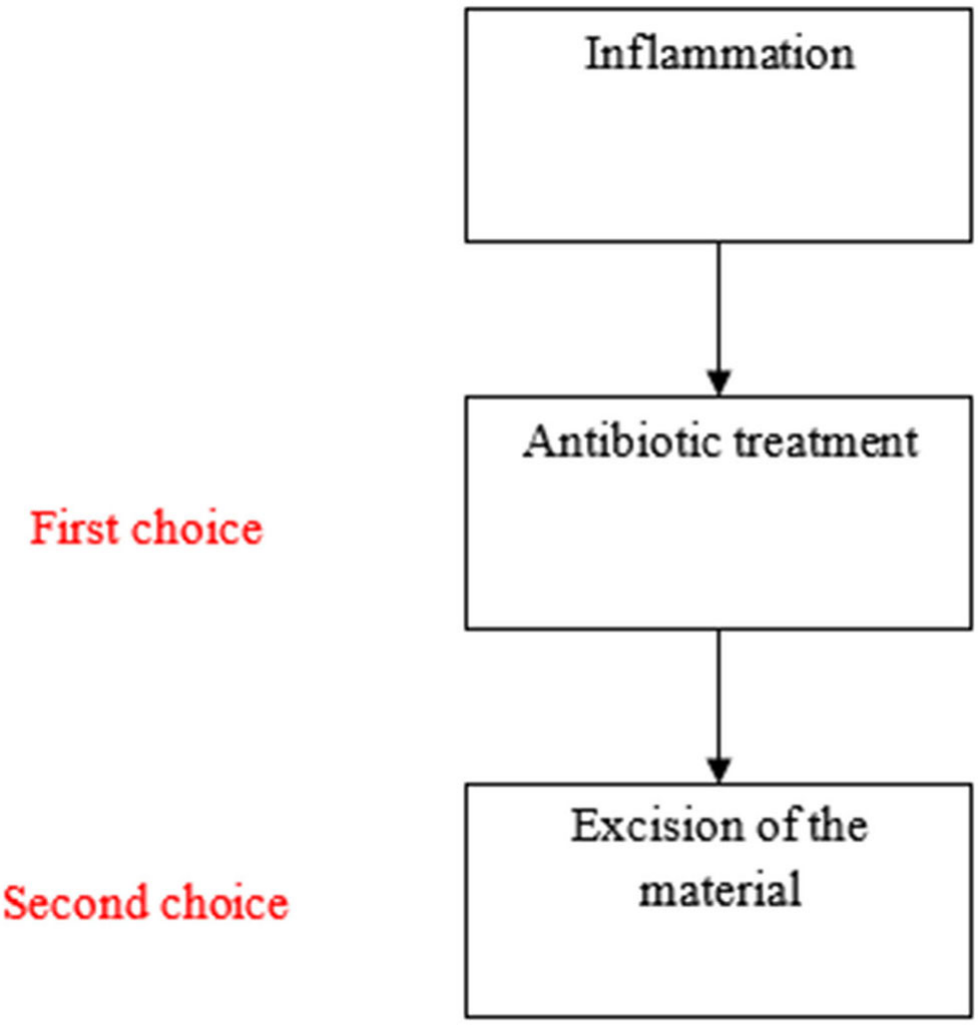
Algorithm for treatment of LIRs

Based on this review, we propose the following treatment algorithm for LIRs (Fig. 4). As stated above, culture proven infections are common after filler injection. Therefore, when clinical features of inflammation such as oedema, heat, erythema, tenderness or pain are present, the first choice should be conservative treatment with oral antibiotics. We recommend the use of tetracyclines because of their dual action as antimicrobial and ant-inflammatory medication. Examples are doxycycline or minocycline (100–200 mg daily). In some cases, non-steroidal anti-inflammatory drugs (NSAIDs) or oral corticosteroids can be added. When conservative treatment fails surgical treatment is required.

## Conclusion

This review has analyzed the aetiopathological hypotheses of LIRs that can currently be found in the literature. A major role is seen for a (pathological) foreign body reaction led by activated histocytes and giant cells, eventually resulting in a chronic inflammation. *S. epidermidis* is the bacterium most often found in LIRs although its role is still debated (contamination and infection, or merely an immunological trigger?). Several substances, such as antibiotics and corticosteroids, seem to be effective in the treatment of LIRs, although the mechanism is not fully understood, and more research is needed.

## Supplementary Information

Below is the link to the electronic supplementary material.

Supplementary file1 (DOCX 32 KB)
