# Supplementary material for: The Aetiopathogenesis of Late Inflammatory Reactions (LIRs) After Soft Tissue Filler Use: A Systematic Review of the Literature
Source: Aesthetic Plast Surg. 2021 Apr 28;45(4):1748–59. doi: 10.1007/s00266-021-02306-3 (PMC8316155; doi:10.1007/s00266-021-02306-3)
Supplement: Supplementary file 1 — Supplementary file1 (DOCX 32 KB) [file 266_2021_2306_MOESM1_ESM.docx]

**Supplemental data file**

**Literature search**

**PubMed 1 August 2019**

[Mesh] = Medical subject headings

[tiab] = words in title or abstract

[ot] = words in 'other terms'-field as author keywords

| **No.** | **Query** | **Results** |
| --- | --- | --- |
| #1 | Search "Morbidity"[Mesh] OR "Mortality"[Mesh] OR "mortality" [Subheading] OR "complications" [Subheading] OR "adverse effects" [Subheading] OR complication*[tiab] OR ((adverse[tiab] OR side[tiab]) AND (effect[tiab] OR effects[tiab] OR event[tiab] OR events[tiab] OR reaction*[tiab])) OR complication*[tiab] OR safety[tiab] OR mortalit*[tiab] OR tolerab*[tiab] OR "adverse effects" [Subheading] OR complication*[ot] OR ((adverse[ot] OR side[ot]) AND (effect[ot] OR effects[ot] OR event[ot] OR events[ot] OR reaction*[ot])) OR complication*[ot] OR safety[ot] OR mortalit*[ot] OR tolerab*[tiab] | 5808214 |
| #2 | Search "CosmoDerm" [Supplementary Concept] OR filler*[ti] OR soft tissue augmentati*[ti] OR soft tissue contour correcti*[ti] OR ((injection*[ti] OR injectable*[ti]) AND silicon*[ti]) OR ((aesthetic*[ti] OR esthetic*[ti] OR dermal*[ti]) AND (microimplant*[ti] OR micro implant*[ti])) OR aesthetic facial contour*[ti] OR esthetic facial contour*[ti] OR filler*[ot] OR soft tissue augmentati*[ot] OR soft tissue contour correcti*[ot] OR ((injection*[ot] OR injectable*[ot]) AND silicon*[ot]) OR aesthetic microimplant*[ot] OR esthetic microimplant*[ot] OR aesthetic micro implant*[ot] OR esthetic micro implant*[ot] OR aesthetic facial contour*[ot] OR esthetic facial contour*[ot] OR ((aesthetic*[ot] OR esthetic*[ot] OR dermal*[ot]) AND (microimplant*[ot] OR micro implant*[ot])) | 4169 |
| #3 | Search (#1 AND #2) | 2207 |

**Embase.com 1 August 2019**

/exp = EMtree keyword with explosion

:ab,ti = words in title or abstract

NEXT/x = words in that order, x places apart

| **No.** | **Query** | **Results** |
| --- | --- | --- |
| #1 | 'injectable implant'/exp OR filler*:ti OR ('soft tissue' NEAR/3 augmentati*):ti OR ('soft tissue contour' NEAR/3 correcti*):ti OR ((injection* OR injectable*) NEAR/3 silicon*):ti OR ((aesthetic* OR esthetic* OR dermal*) NEAR/3 (microimplant* OR 'micro implant' OR 'micro implants')):ti OR ('aesthetic facial' NEAR/3 contour*):ti OR ('esthetic facial' NEAR/3 contour*):ti | 4678 |
| #2 | 'morbidity'/de OR 'mortality'/de OR 'surgical mortality'/exp OR 'complication'/exp OR 'side effect'/exp OR 'safety'/de OR complication*:ab,ti OR ((adverse OR side) NEAR/4 (effect OR effects OR event OR events OR reaction*)):ab,ti OR safety:ab,ti OR mortalit*:ab,ti OR tolerab*:ab,ti | 4185379 |
| #3 | #1 AND #2 | 1807 |

**Wiley/Cochrane Library 1 August 2019**

ti,ab,kw = words in title, abstract or keyword

| **No.** | **Query** | **Results** |
| --- | --- | --- |
| #1 | filler* or ("soft tissue" and augmentati*) or ("soft tissue" and contour and correcti*) or ((injection* or injectable*) and silicon*) or ((aesthetic* or esthetic* or dermal*) and (microimplant* or "micro implant" or "micro implants")) or ((aesthetic or esthetic) and facial and contour*):ti,ab,kw (Word variations have been searched) | 810 |
| #2 | complication* or ((adverse or side) and (effect or effects or event or events or reaction*)) or safety or mortalit* or tolerab*:ti,ab,kw (Word variations have been searched) | 329566 |
| #3 | #1 and #2 | 315 |

**Table 6.** Human studies, local and systemic immunological factors found in dermal implant material or adjacent tissue

| **Groups** | **N** | **Primary indication** | **Type of complication** | **Follow-up, injection duration**  **Mean (range)** | **Immunological detection method** | **Immunological factor** | **Results** | **Conclusion** | **Author,**  **year of publication** |
| --- | --- | --- | --- | --- | --- | --- | --- | --- | --- |
| No groups | 7 | Unknown | Intermediate or late onset side effects | 65 months (6-114 months) | Laboratory testing +unknown histopathologic examination | Histopathologic examination | Fibrinogen/ CRP (7/7 increase), LDH (5/7 increase), ACE (4/7 increase), Complement pathway proteins 3 and 4 (decrease 3/7), Antinuclear antibodies (5/7 positive), AAENA (5/5 negative), SPRE (6/7 elevated), T-helper/t-cytotoxic ratio (2/3 elevated) | According to our results, tracrolimus seems to be an effective drug in the medical management of late-onset, inflammatory, immune-mediated adverse effects related to SFI that are refractory to usual therapy, that is, nonresponders, even when they have been administered together. | Alijotas-Reig, J *et al*. 2012 |
| No groups | 33* | Unknown | Unknown | 7 days - 3 years (mean 14 days for Aquamid), others unknown | Light microscope + HE-stain, periodic acid-Schiff-stain, and Gram-stain. | Histopathologic examination | Heavy foreign body reactions were present in most biopsies. | Both intermediate and long-term adverse reactions are caused by a bacterial low-grade infection, and inherent characteristics of the implant determine the development of associated fibrosis. | Christensen, L *et al.* 2005 |
| No groups | 8 | Unknown | Granuloma's | Unknown | Light microscope, polarized light microscope + HE-stain. Electron microscope for energy dispersive x-ray elemental microanalysis | None | Filler lesions consisted of granulomatous inflammation with epithelioid histocytes and giant cells. Also, a high concentration of calcium and phosphorous was detected | Granulomatous inflammation can be a foreign body-reaction after injection with Radiesse | Daley, T  *et al.* 2012 |
| Complication groups: acute (<6 months) vs chronic (>6 months) | 96 | Unknown | Acute/late complications/ abscess/ hardened nodules | Unknown | Unknown | None | Inflammatory characteristics were found in filler lesions, as well as PMMA microspheres | Complications are related to an inflammatory process that, in the late phase, produces hardened nodules. | de Melo Carpaneda, E  *et al.* 2012 |
| No groups | 38 | Unknown | Indurated plaque/nodular lesion/ inflammatory mass/ atrophic lesion/skin discoloration/ ulceration | 6-25 months 14.6 months (6-25 months) | Polarized light microscope + HE-stain, 'special stains' | None | 4 types of histologic patterns of filler complications were found next to foreign body granuloma: chronic inflammation, fibrosis, fat necrosis and panniculitis | Various histological reactions to dermal fillers exist. | El-Khalawany, M *et al.* 2015 |
| No groups | 12 | Unknown | Submucosal/ nodules/ swelling | Unknown | immunoperoxidase staining | CD68, cytokeratins AE1,AE2 and S-100 protein expression | Morphologic patterns are distinguished by filler type. Four different histopathologic responses could be differentiated: nodule without foreign body reaction, nodule with chronic inflammation, granuloma with epithelioid histiocytic and multinucleated giant cell reaction | Variations in morphology and host response to dermal fillers are unique for each substance | Eversole, R  *et al.* 2013 |
| No groups | 7 | Esthetic (n=6), traumatic (n=1) | Granuloma's | Unknown | Light microscope + HE-stain | Fibrosis/histocytes and lymphocytes | Unknown | Unknown | Ficarra, G  *et al.* 2012 |
| No groups | 19 | Facial lipoatrophy (n=4), esthetic (n=15) | (Non)inflammatory nodules, abscess | Unknown | HE-stain. Detection of plasmacytoid dendritic cells using immunohistochemical staining. | CD123, Anti-CD11c | No pDCs detected in 8 of 10 polyalkylimide gel, in 1 of 2 polyacrylamide gel, and the 5 liquid injectable silicone biopsies. All 4 HEMA/EMA biopsies contained collections of pDCs in lymphocytic infiltrates close to filler particles and adjacent sarcoidal granulomas. | Plasmacytoid dendritic cells may contribute to sarcoidal granulomas associated with injected GEMA/EMA | Kadouch, JA  *et al.* 2015 |
| No groups | 11 | Cosmetic | Granuloma's | Unknown | Unknown | None | Three kinds of classic foreign-body granulomatype (featuring numerous giant cells around foreign bodies) were distinguished. A cystic and macrophagic granuloma type was found, characterized by extracellular microcysts, surrounded by a mainly mononuclear infiltrate of vacuolated macrophages | The morphologic aspect of the foreign particles allow to identify the injected product | Lombardi, T  *et al.* 2004 |
| No groups | 8 | Unknown | Redness, pruritus, painful swelling and nettle-type rash | Up to 4.5 months | Lymphocyte transforming test, intradermal test and serum test | None | The skin tests  were positive for one or the other or both of the injectable hyaluronic acid preparations. The different biopsies have shown for some a chronic inflammatory reaction, even 11 months after the treatment or a severe granulomatous reaction to foreign bodies. Serum analysis revealed positive antibodies against Restylane and/or Hylaform and even IgG and E anti-hyaluronic acid | I have 8 patients with adverse reactions to injectable hyaluronic acid, which after several tests, may be allergic to those products | Micheels, P  *et al.* 2001 |
| 1. Consistent with hyaluronic acid 2. Consistent with calcium hydroxyapatite 3. Consistent with Poly-L-lactic acid 4. Consistent with polyacrylamide gel 5. Consistent with liquid | 16 | Unknown | Granuloma's | Unknown | Microscope + HE-stain, infrared spectrometer, Raman spectrometer | None | With the help of spectrometric analysis, we were able to correlate the histopathologic presentations with the specific  type of dermal filler used | Different histopathologic presentations of dermal filler foreign body reactions can be correlated with the specific type of dermal filler used. | Owosho, A *et al.* 2014 |
| No groups | 5 | Unknown | Granuloma's | Unknown | HE-stain | None | Bioplastique granuloma presents with irregularly shaped cystic spaces of varying size containing jagged, translucent, no birefringent foreign bodies. Artecoll granuloma shows numerous round vacuoles nearly identical in size and shape enclosing round and sharply circumscribed, translucent, nonbirefringent foreign bodies | The distinctive histopathologic findings of each microimplant will lead to the correct diagnosis. | Rudolph, C  *et al.* 1999 |
| No groups | 25 | Unknown | Granuloma's | 6.5 months (2- 36 months) | Polarized light microscope + HE-stain | CD163 | The regularly sized mauve-to-gray or beige spherules of CHA measured 20 to 40 μm. The ovoid, rice-shaped, or spindle-shaped refractile structures of PLA measured 50 to 150 μm in length and 15 to 35 μm in width. The granulomas associated with PLA may contain asteroid bodies. | CHA and PLA produce granulomatous inflammation with distinct and readily recognized features. | Shahrabi-Farahani, S  *et al.* 2014 |
| No groups | 10 | Unknown | Inflammatory nodules | Up to 8 years | Microscope, electron microscope | None | Multinucleated foreign-body giant cells were found. Epithelioid cells, macrophages, plasma cells, and a more or less sparse lymphocytic infiltrate were found surrounding | Inflammatory nodules due to adverse reactions to permanent fillers containing microparticles  with a hydrophobic surface were treated with good results with a regimen of allopurinol and  intralesional injections with a mixture of fluorouracil and low-dose triamcinolon. | Wiest, L  *et al.* 2009 |

*Biopsies

**Table 7.** Human and animal studies, bacterial contamination found in dermal implant material or adjacent tissue

| **Groups** | **N** | **Primary indication** | **Type of complication** | **Follow-up/ implant duration Mean (range)** | **Microbiological detection method** | **Results** | **Conclusion** | **Author, year of publication** |
| --- | --- | --- | --- | --- | --- | --- | --- | --- |
| No groups | 7 | Unknown | Intermediate or late onset side effects | 65 months (6-114 months) | Unknown | No microorganisms were observed | Unknown regarding microbiology | Alijotas-Reig, J *et al*. 2012 |
| No groups | 8 | Unknown | Unknown | Unknown (5 months - 2.5 years) | Light microscope + epi-fluorescence microscope | Bacteria were found in filler lesions, lying in clusters. The vast  majority of bacteria were cocci. We also performed  PNA FISH with a PNA probe targeting coagulasenegative staphylococci, because these bacteria have  been found to be common in infections with solid  implants,6 but only one of the biopsies was positive  for these bacteria. | Extreme care must be taken when injecting a permanent filler. Profylactic antibiotics are recommended. | Bjarnsholt, T *et al.* 2009 |
| Grade 1 (n=13), grade 2 (n=19), grade 3 (n=21), grade 4 (n=6), control (n=28) | 74 | Unknown | Unknown | 492 days (9-2161 days) | Confocal laser scanning microscopy, FISH-analyses | A significant increase in the incidence of Staphylococcus epidermidis was detected in  the control group (P=0.000) compared to the study group. The study group showed a significantly  higher incidence of Staphylococcus aureus (P=0.005), Klebsiella pneumoniae (P=0.006),  Klebsiella oxytoca (P=0.048), and Staphylococcus haemolyticus (P=0.048) compared to the  control group. | The bacterial flora on the skin differed in patients with LBI from the control  group. The control group’s bacterial skin flora was dominated by S. epidermidis. Patients with  LBI had a bacterial skin flora dominated by potentially pathogenic bacteria | Christensen, L *et al.* 2013 |
| No groups | 33* | Unknown | Unknown | 7 days - 3 years (mean 14 days for Aquamid), others unknown | Culture swab and PCR/DNA cloning | Yes in all Aquamid patients, in 1 out of 2 Dermalive patients, none of the New-Fill patients. | Both intermediate and long-term adverse reactions are caused by a bacterial low-grade infection, and inherent characteristics of the implant determine the development of associated fibrosis. | Christensen, L *et al.* 2005 |
| No groups | 24 | Esthetic | Low-grade inflammation or abscess | 38 months  (1 mo- 10 years) | Cultures | Positive cultures identified Staphylococcus aureus (n = 13) as the predominant pathogen, followed by Enterobacter aerogenes (n = 2), Streptococcus sanguinis (n = 1), Pseudomonas aeruginosa (n = 1), Escherichia coli (n = 1), and Streptococcus agalactiae (n = 1). | The possibility of bacterial contamination of a filler depot during an invasive treatment poses a problem for the use of permanent fillers. | Kadouch, J *et al. 2013* |
| 1: Complication group (n= 10 patients, n= 40 swabs),  2: Healthy control group (n=17 patients, n= 51 swabs) | 27 | Esthetic | Late bacterial infections | 5.2 months  (1-18 months) | Culture swab  Columbia/ mannitol salt/ MacConkey/ chocolate/Schaedler agar | A significant increase in the incidence of Staphylococcus epidermidis was detected in  the control group (P=0.000) compared to the study group. The study group showed a significantly  higher incidence of Staphylococcus aureus (P=0.005), Klebsiella pneumoniae (P=0.006),  Klebsiella oxytoca (P=0.048), and Staphylococcus haemolyticus (P=0.048) compared to the  control group. | The bacterial flora on the skin differed in patients with LBI from the control  group. The control group’s bacterial skin flora was dominated by S. epidermidis. Patients with  LBI had a bacterial skin flora dominated by potentially pathogenic bacteria. | Netsvyetayeva, I *et al*. 2018 |
| No groups | 5 | Unknown | Unknown | Unknown | Culture and PCR | Microbiome analysis detected a  predominance of Pseudomonas, Staphylococcus, and Propionibacterium as present  in these samples. | Biofilm appears to be associated with high numbers in clinical samples  of patients presenting with chronic granulomatous inflammation. | Saththianathan, M  *et al.* 2017 |
| No groups | 10 | Unknown | Unknown | Up to 8 years | Unknown | No microorganisms were observed | Unknown regarding microbiology | Wiest, L  *et al.* 2009 |

**Table 8a.** Human and animal studies, anti-inflammatory therapy

| **Groups** | **N** | **Primary indication** | **Type of complication** | **Follow-up**  **Mean (range)** | **Anti-inflammatory drug** | **Results** | **Conclusion** | **Author, year of publication** |
| --- | --- | --- | --- | --- | --- | --- | --- | --- |
| No groups | 7 | Unknown | Intermediate or late onset side effects | 65 months (6-114 mo) | Tacrolumus (up to 0.08/0.1 mg/kg) | Clinical response was good and achieved promptly, usually within 1 to 2 weeks. | According to our results, tracrolimus seems to be an effective drug in the medical management of late-onset, inflammatory, immune-mediated adverse effects related to SFI that are refractory to usual therapy, that is, nonresponders, even when they have been administered together | Alijotas-Reig, J *et al*. 2012 |
| Complication groups: acute (<6 months) vs chronic (>6 months) | 96 | Unknown | Acute/late complications/ abscess/ hardened nodules | Unknown | Local corticosteroid injection | These patients reported an initial recovery with the  softening of the compromised region but after a few  months the nodule became harder and the skin of the region  where the product had been injected turned whitish and  depressed | Complications are related to an inflammatory process that, in the late phase, produces hardened nodules. | de Melo Carpaneda, E et al.  2012 |
| 1: prophylactic AB treatment  8 mice + 8 control  2: AB treatment post-surgery 8 mice + 8 control.  3: Triamcinolone treatment post-surgery  8 mice + 8 control | 48 | Experiment | n/a | 0 hours- 7 days | Triamcinolone Acetonide | Evaluation of  treatment strategies showed that once the bacteria had settled (into biofilms) within  the gels, even successive treatments with high concentrations of relevant antibiotics/ corticosteroids  were not effective. | Treatment with AB and/or steroids do not have effects. However, profylactic use seems to have effect. | Alhede, M *et al.*  2014 |

*Biopsies

**Table 8b.** Human and animal studies, type of systemic antibiotics

| **Groups** | **N** | **Primary indication** | **Type of complication** | **Antibiotics/ antimicrobial** | **Follow-up Mean (range)** | **Results** | **Conclusion** | **Author, year of publication** |
| --- | --- | --- | --- | --- | --- | --- | --- | --- |
| No groups | 8 | Unknown | Unknown | penicillin, fluoroquinolone, cephalosporin | Unknown (5 months - 2.5 years) | Unknown regarding antibiotics | Extreme care must be taken when injecting a permanent filler. Profylactic antibiotics are recommended. | Bjarnsholt, T *et al.* 2009 |
| Group 1: AB first option (n=5). Group 2: AB second option (n=12).  Group 3: other treatments (n=5) | 22 | Unknown | Late bacterial infections | Moxifloxacin (2x400 mg per os) and clarithromycin (2x500 mg per os) | 3.59 months (1-18 monts) | Of the 17 patients cured with the AB scheme, 5 were cured after application of the scheme as the first therapeutic option, whereas for 12 it was applied only after unsuccessful treatment with other schemes | The AB scheme is recommended as the first therapeutic option for treating LBI complications related to soft-tissue fillers | Marusza, W *et al.* 2019 |
| 1: prophylactic AB treatment  8 mice + 8 control  2: AB treatment post-surgery 8 mice + 8 control.  3: Triamcinolone treatment post-surgery  8 mice + 8 control | 48 | Experimental | n/a | Tobramycin or rifampicin | 0 hours- 7 days | Evaluation of  treatment strategies showed that once the bacteria had settled (into biofilms) within  the gels, even successive treatments with high concentrations of relevant antibiotics  were not effective. | Treatment with AB and/or steroids do not have effects. However, prophylactic use seems to have effect | Alhede, M *et al.* 2014 |
